# Healthcare Professionals’ Preferred Efficacy Endpoints and Minimal Clinically Important Differences in the Assessment of New Medicines for Chronic Obstructive Pulmonary Disease

**DOI:** 10.3389/fphar.2019.01519

**Published:** 2020-02-06

**Authors:** Marloes Dankers, Marjorie H. J. M. G. Nelissen-Vrancken, Sara M. K. Surminski, Anke C. Lambooij, Tjard R. Schermer, Liset van Dijk

**Affiliations:** ^1^Dutch Institute for Rational Use of Medicine, Utrecht, Netherlands; ^2^Nivel Netherlands Institute for Health Services Research, Utrecht, Netherlands; ^3^Department of Primary and Community Care, Radboud Institute for Health Sciences, Radboud University Medical Center, Nijmegen, Netherlands; ^4^Department of Pharmacotherapy, Pharmacoepidemiology and Pharmacoeconomics (PTEE), Faculty of Mathematics and Natural Sciences, Groningen Research Institute of Pharmacy, University of Groningen, Groningen, Netherlands

**Keywords:** clinical relevance, minimal clinically important differences, patient-reported outcomes, chronic obstructive pulmonary disease, new medicine, forced expiratory volume in 1 sec, St. George’s Respiratory Questionnaire, Transition Dyspnea Index

## Abstract

**Background:**

Registration authorities evaluate effects of new medicines for chronic obstructive pulmonary disease (COPD) on airway obstruction, dyspnea, health status and exacerbations. To establish clinical relevance, minimal clinically important differences (MCIDs) are used. The aim of this study was to investigate which efficacy endpoints and MCIDs healthcare professionals consider clinically relevant for new COPD medicines.

**Materials and Methods:**

7,731 Healthcare professionals received an electronic questionnaire. Participants were asked for: 1) preferred efficacy endpoints for new COPD medicines and 2) cut-off values defining clinical relevance for forced expiratory volume in 1 sec (FEV_1_), Transition Dyspnea Index (TDI) and St. George’s Respiratory Questionnaire (SGRQ). Those cut-off values were compared to the MCIDs used by registration authorities, namely 100 ml for FEV_1_, 1 unit for TDI and 4 units for SGRQ.

**Results:**

227 Healthcare professionals responded to the questionnaire. Most preferred efficacy endpoints were exacerbations (51.0%), airway obstruction (46.9%) and health status (44.9%). Mean cut-off values for TDI and SGRQ were significantly higher than the corresponding MCIDs, mean differences 1.5 (95%CI = 1.3–1.8, p < 0.001) and 7.0 (95%CI = 5.1–8.8, p < 0.001), respectively. The mean cut-off value for FEV_1_ was comparable to the MCID (mean difference 2.2, 95%CI = -19.9–24.3, p = 0.84).

**Conclusions:**

Healthcare professionals largely agree with efficacy endpoints used for the evaluation of new COPD medicines. However, they seem to prefer higher cut-off values for clinical relevance for TDI and SGRQ than the registration authorities. Effects of new medicines on TDI and SGRQ that are considered clinically relevant by registration authorities do, therefore, not necessarily reflect healthcare professionals’ perspectives on clinical relevance.

## Introduction

New medicines are evaluated by registration authorities in order to assess their benefit-risk balance. The assessment of clinical relevance of new medicines by the registration authorities depends heavily on clinical trials. A common approach in clinical trials to investigate clinical effects is the use of patient-reported outcomes (PROs) (Hedayat et al., 2015). PROs represent the patient perspective, quantifying the extent to which a disease impacts their health and functioning (Jones et al., 2012). To establish the clinical relevance of a specific improvement on an endpoint minimal clinically important differences (MCIDs) are used. An MCID is the smallest difference which patients perceive as beneficial and which would mandate a change in patient treatment (Donohue, 2005). Establishing whether an improvement on a clinical endpoint exceeds the MCID is a way to evaluate the clinical relevance of a (new) pharmacological treatment (Make, 2007; Koynova et al., 2013; Bartels et al., 2017).

In the last decade, multiple new medicines for the treatment of chronic obstructive pulmonary disease (COPD) obtained market access (CHMP, 2009; CHMP, 2012a; CHMP, 2012b; CHMP, 2014). Assessment of new COPD medicines includes the evaluation of effects on several efficacy endpoints, including airway obstruction, dyspnea, health status, and exacerbations. Frequently used parameters are forced expiratory volume in 1 sec (FEV_1_), Transition Dyspnea Index (TDI) and St. George’s Respiratory Questionnaire (SGRQ) for airway obstruction, dyspnea and health status respectively (CHMP, 2009; CHMP, 2012a; CHMP, 2012b; CHMP, 2012c; CHMP, 2014). Although FEV_1_ is an objective endpoint and highly reproducible (Cazzola et al., 2008), it has a relatively poor correlation with symptoms (Jones et al., 2012; Singh, 2017). PROs like dyspnea and health status might better reflect the impact of the disease on COPD patient’s daily life. Improvements of 100 ml, one units and four units are validated MCIDs for FEV_1_, TDI, and SGRQ, respectively (Donohue, 2005; Make, 2007; Cazzola et al., 2008; Jones et al., 2014). Although these values are widely adopted, some debate about the acceptability of these MCIDs exists, especially for FEV_1_ (Donohue, 2005). Values up to 140 ml are suggested as MCID for FEV_1_ (Cazzola et al., 2008). There is no clear MCID for the evaluation of exacerbations (Chapman et al., 2013).

Since FEV_1_, TDI, and SGRQ and their corresponding MCIDs are used by registration authorities for the evaluation of clinical efficacy of new medicines for the treatment of COPD, they are of crucial importance for the market access of these new medicines. Physicians and other healthcare professionals who prescribe (or advise about) those medicines have to rely on the assessment of new medicines by registration authorities. It is therefore of particular interest to know their opinions about the endpoints and MCIDs used in the assessment of clinical relevance of new medicines. Although expert-opinions can be included in the establishment of an MCID (in addition to the use of statistical and anchor-based approaches) (Beaton et al., 2002; Make, 2007), it is to our knowledge unknown how healthcare professionals assess the clinical importance of endpoints and their MCIDs used for new COPD medicines. The aims of this study are therefore: 1) to investigate which efficacy endpoints healthcare professionals consider clinically relevant in the assessment of new medicines for COPD, and 2) to investigate which MCIDs healthcare professionals consider relevant for the frequently used endpoints FEV_1_, TDI, and SGRQ.

## Materials and Methods

### Background

Airway obstruction (measured by FEV_1_), dyspnea (measured by TDI), and health status (measured by SGRQ) are important efficacy endpoints in the assessment of new medicines for COPD (CHMP, 2012c). FEV_1_ is the volume of air that is forcibly exhaled in the 1st second. The trough (pre-bronchodilator) FEV_1_ is most commonly used in clinical trials evaluating the efficacy of COPD medicines (Donohue, 2005). A commonly used MCID for trough FEV_1_ is 100 ml (Donohue, 2005; Make, 2007; Cazzola et al., 2008; Jones et al., 2014). TDI is a validated evaluative instrument that measures changes in the severity of dyspnea by grading functional impairment, magnitude of task and magnitude of effort. Each parameter is graded from -3 to +3, adding up to a total score ranging from -9 to +9 (Cazzola et al., 2008). The MCID is 1 unit (Donohue, 2005; Make, 2007; Cazzola et al., 2008; Jones et al., 2014). SGRQ has been developed to measure health status in patients with respiratory disease. It is a self-administered questionnaire that measures health status in the subdomains symptoms, activity and impacts, with a total score ranging from 0 to 100 (Cazzola et al., 2008; Jones et al., 2012). A difference of four units is considered clinically relevant (Donohue, 2005; Make, 2007; Cazzola et al., 2008; Jones et al., 2014).

### Design

This investigation was part of a more extensive online survey about the opinion of Dutch healthcare professionals regarding the clinical relevance of new medicines for the treatment of diabetes mellitus type 2 (T2DM) and COPD. No ethical approval was needed. According to the Dutch legislation, neither obtaining informed consent nor approval by a medical ethics committee is obligatory for carrying out research among healthcare professionals that does not include patient data.

### Participants

Participants for the online survey were obtained from the Customer relationship management (CRM) of the Dutch Institute for Rational Use of Medicine (IRUM). This CRM has multiple purposes, but is predominantly used for sending newsletters and information about the IRUMs activities. The CRM contained 7,731 email addresses of Dutch healthcare professionals (predominantly physicians, pharmacists, practice nurses, respiratory nurses, and diabetes nurses).

### Data Collection

The invitation to fill out the questionnaire was sent by email (with a link to the questionnaire) on 15 November 2016. The online survey was closed two weeks later. All healthcare professionals received one reminder after 1 week. Participants did not receive a financial compensation, although every 10^th^ participant was offered a free online accredited medicine course.

### Questionnaire and Measurements

The full questionnaire consisted of 39 questions, among them 19 questions about new medicines for COPD. Responders were first asked for their profession. Next, they were asked whether they were involved in the management of patients with T2DM or COPD in their daily clinical practice. Only healthcare professionals working with COPD patients were asked to fill out the COPD section of the questionnaire.

The content of the COPD section of the questionnaire was based on the requirements for clinical trials for new COPD medicines, as described in the *Guideline on clinical investigation of medicinal products in the treatment of COPD* (CHMP, 2012c) and the MCIDs mentioned in the public assessment reports of new medicines for COPD (CHMP, 2009; CHMP, 2012a; CHMP, 2012b; CHMP, 2014).

The COPD section of the questionnaire consisted of three parts. The first part investigated the healthcare professionals preferred efficacy endpoints for the assessment of clinical relevance of new COPD medicines. Healthcare professionals were first informed about the need of efficacy endpoints in clinical trials and then asked which efficacy endpoints they considered clinically relevant for the assessment of new COPD medicines. All questions were open-ended in order to enhance the chance of getting reliable and sincere answers.

The second part investigated cut-off values for clinical relevance for FEV_1_, TDI, and SGRQ. Healthcare professionals were informed about these endpoints and asked for a cut-off value for clinical relevance compared to placebo. All questions were open-ended. The MCIDs for FEV_1_, TDI, and SGRQ were not mentioned before, in order to stimulate healthcare professionals to base their answer on their own clinical experience without being influenced by the validated MCIDs. Since TDI and SGRQ are not routinely used in daily clinical practice in the Netherlands, the questionnaire gave a brief description of these endpoints, including the range of those scales. This enabled responders to estimate a clinically relevant difference, regardless of their familiarity with those parameters. The third part about safety endpoints was not included in this analysis.

The online survey was programmed in NetQuestionnaire and pre-tested by three general practitioners, two pharmacists and three practice nurses for reasons of understandability and content. Since they experienced more difficulties with the COPD section compared to the T2DM section, the T2DM section was positioned before the COPD section to enhance the overall response rate.

### Data Analysis

Responders were categorized by profession (physician, pharmacist, practice nurse, and other). The group “other” was excluded from further analysis. Responders who were both a physician and pharmacist were analyzed as physicians. All different types of nurses (for example, practice nurses, respiratory nurses, and nurse practitioners) were analyzed together as practice nurses.

After collection of the endpoints mentioned by the responders, six different categories of endpoints based on Global strategy for prevention, diagnosis and management of COPD 2019 report [Global Initiative for Chronic Obstructive Lung Disease (GOLD), 2019] were defined. Those categories were 1) exacerbations (including hospital admissions, infections, use of antibiotics and oral steroids), 2) airway obstruction (including parameters used to define airway obstruction like FEV_1_), 3) health status (including quality of life, disease burden, Clinical COPD Questionnaire (CCQ) wellbeing and daily functioning), 4) respiratory symptoms (including dyspnea, cough, Medical Research Council (MRC) dyspnea scale, use of short-acting bronchodilators), 5) exercise intolerance (including condition, physical activity), 6) mortality, and 7) other (including morbidity, oxygen dependency or saturation, and adverse events). Subsequently, all answers were categorized by two researchers (based on consensus) and frequencies were calculated. Some healthcare professionals mentioned the Global Initiative for Chronic Obstructive Lung Disease (GOLD) status as preferred endpoint. Since GOLD includes airway obstruction, exacerbations and health status [Global Initiative for Chronic Obstructive Lung Disease (GOLD), 2019], each answer that mentioned GOLD was counted into these three categories. The categorization was verified by one independent researcher.

All cut-off values in open text fields were recoded into numeric variables. Impossible values for TDI and SGRQ (i.e., values exceeding the parameter range) were excluded. Based on expert opinions, FEV_1_ values > 0 and < 1 ml and FEV_1_ values > 1,499 ml were considered implausible and therefore also excluded, as were values in other units than asked (e.g., percentages instead of milliliters). Ranges (e.g., 50–75) were converted to averages (e.g., 67.5). Cut-off values for FEV_1_, TDI and SGRQ were compared to the corresponding MCIDs by a one-sample T-test. Results were considered significant when p < 0.05.

All results were analyzed with IBM SPSS Statistics 24.

## Results

**Figure 1:**
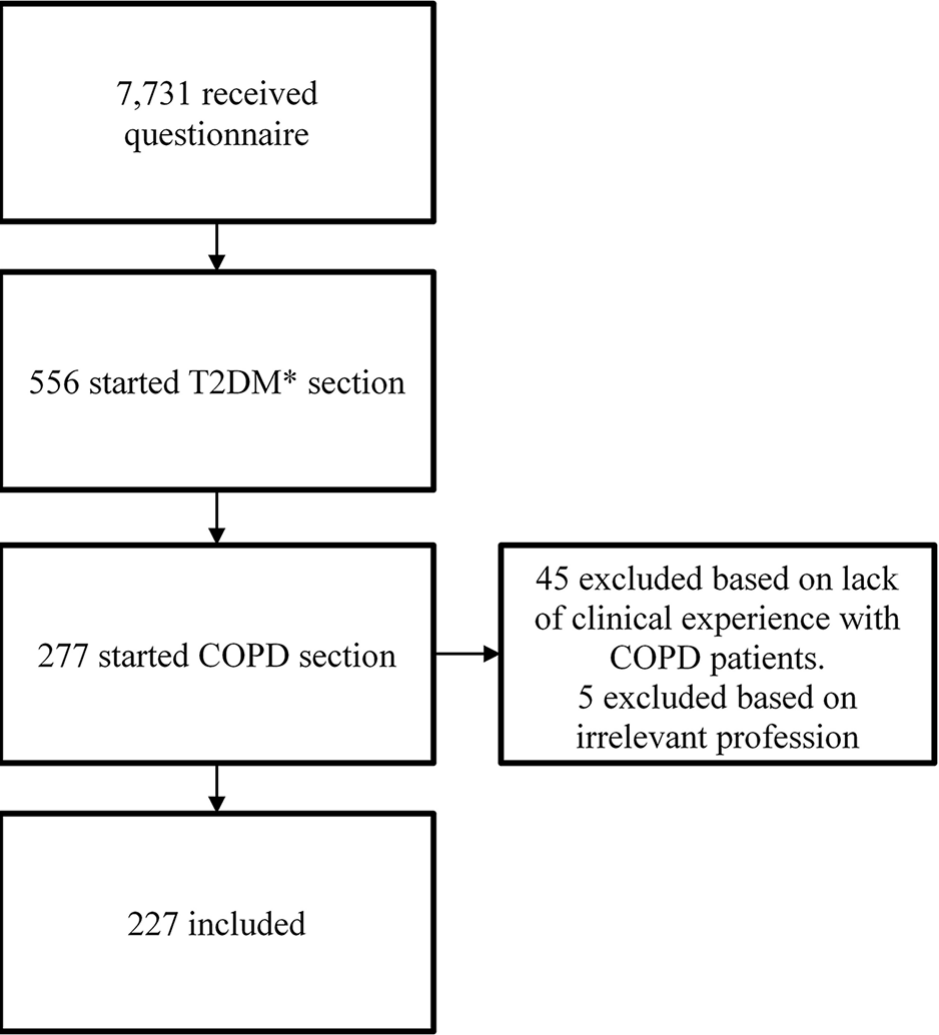
Response. *T2DM = diabetes mellitus type 2.

A total of 556 responders (6.6%) started the questionnaire. Only healthcare professionals working with COPD patients in daily practice were included in this analysis. The final population consisted of 227 healthcare professionals (88 physicians, 107 pharmacists, and 27 practice nurses), resulting in a response rate of 2.9% (**Figure 1**). The group of 88 physicians contained two physicians who were also pharmacists and one dispensing general practitioner. Those were analyzed solely as physicians. Among the group of 27 practice nurses were 21 practice nurses, one respiratory nurse, one physician assistant, one nurse practitioner, one trainee nurse practitioner, one geriatric nurse, and one practice nurse who was also a respiratory nurse and diabetes nurse. Those were analyzed together as practice nurses.

### Endpoints

196 healthcare professionals mentioned their efficacy endpoints of preference. The most frequently mentioned endpoints were exacerbations (51.0%), airway obstruction (46.9%), and health status (44.9%) (**Table 1**).

**Table 1:** Preferred efficacy endpoints in the evaluation of new COPD medicines.

Endpoints	Frequency
Exacerbations	51.0%
Airway obstruction*	46.9%
Health status	44.9%
Respiratory symptoms	30.6%
Mortality	23.0%
Exercise intolerance	9.2%
Other	11.2%

*2 responders (1%) mentioned inspiratory capacity as most preferred endpoint. Since this is closely related to airway obstruction, those answers were counted into this category.

### MCIDs

Healthcare professionals were asked for a cut-off value that defined clinical relevance for FEV_1_, TDI, and SGRQ. For both TDI and SGRQ, cut-off values according to healthcare professionals (2.5 and 11.0 units, respectively) were significantly higher than the MCIDs used by the European Medicines Agency (EMA) (one unit and four units, respectively) (**Table 2**). Mean differences for TDI and SGRQ compared to MCID’s were 1.5 (95% CI = 1.3–1.8, p < 0.001) and 7.0 (95% CI = 5.1–8.8, p < 0.001). Pharmacists mentioned the highest cut-off values for both TDI (2.8 units; followed by 2.4 units by physicians and 2.2 units by practice nurses) and SGRQ (12.2 units, followed by 11.5 units by practice nurses and 9.5 units by physicians).

**Table 2:** Cut-off values for FEV_1_, TDI, and SGRQ according to healthcare professionals, compared to the corresponding MCID.

		n	Mean (SD)	Mean difference compared to MCID (95%CI)	p
FEV_1_ (ml)	**All**	**105**	**102.2 (114.1)**	**2.2 (-19.9–24.3)**	**0.84**
MCID = 100 ml	Physicians	44	115.3 (108.8)	15.3 (-17.8–48.3)	0.36
	Pharmacists	44	100.8 (133.6)	0.75 (-39.9–41.4)	0.97
	Practice nurses	17	72.1 (57.8)	-27.9 (-57.6–1.9)	0.064
TDI (unit)	**All**	**109**	**2.5 (1.4)**	**1.5 (1.3–1.8)**	<0.001*
MCID = 1 unit	Physicians	44	2.4 (1.3)	1.4 (1.0–1.7)	<0.001*
	Pharmacists	53	2.8 (1.5)	1.8 (1.4–2.2)	<0.001*
	Practice nurses	12	2.2 (1.6)	1.2 (0.16–2.2)	0.027*
SGRQ (unit)	**All**	**119**	**11.0 (10.1)**	**7.0 (5.1–8.8)**	<0.001*
MCID = 4 units	Physicians	50	9.5 (6.4)	5.5 (3.7–7.3)	<0.001*
	Pharmacists	56	12.2 (11.9)	8.2 (5.0–11.3)	<0.001*
	Practice nurses	13	11.5 (13.4)	7.5 (-0.56–15.6)	0.065

* Statistically significant different compared to MCID, based on one-sample T-test. Results were considered significant when p < 0.05.

The mean cut-off value for FEV_1_ (102.2 ml) according to healthcare professionals was comparable to the MCID (100 ml), mean difference 2.2 ml (95% CI = -19.9–24.3, p = 0.84). Mean cut-off values according to pharmacists, physicians and practice nurses were 100.8 ml, 115.3 ml, and 72.1 ml, respectively. None of these cut-off values were significantly different from the MCID.

## Discussion

This study investigated which endpoints and MCIDs healthcare professionals considered clinically relevant for the evaluation of the efficacy of new medicines for COPD. Dutch healthcare professionals seem slightly more critical than registration authorities in the assessment of the clinical relevance of those new medicines. Although the preferred endpoints roughly correspond with the ones used in clinical trials, healthcare professionals prefer higher cut-off values for clinical relevance for TDI and SGRQ than the MCIDs used by registration authorities. This stricter view of clinical relevance is not seen for airway obstruction, since the average cut-off value for FEV_1_ was comparable to the MCID. In general, physicians and practice nurses were less critical than pharmacists. This may display a difference in the clinical experience of healthcare professionals. Practice nurses and physicians more often see patients and measure endpoints like airway obstruction, dyspnea and health status than pharmacists. That might enhance their ability of estimating the expected medicine-induced improvement.

To our knowledge, this is the first study that specifically investigated the opinions of healthcare professionals about the endpoints and MCIDs used for the assessment of clinical efficacy of new COPD medicines. Expert opinions on the MCID for FEV_1_ have, however, been published before. The cut-off values for FEV_1_ according to a small group of opinion leaders on this topic were generally higher than the MCID of 100 ml and thus also higher than the cut-off value for FEV_1_ found in our investigation (Donohue, 2005).

The differences between the cut-off values found in this investigation and established MCIDs might reflect the challenges with MCIDs as stated in other publications (Donohue, 2005; Kiley et al., 2005; Jones et al., 2014). Factors such as heterogeneity in population and disease, trial duration, Hawthorne effects, withdrawal rates, and baseline disease severity may affect the size of benefit relative to the MCID (Kiley et al., 2005; Jones et al., 2014). It is therefore suggested that MCIDs should be used as an indicative value rather than as an absolute cut-off point (Jones et al., 2014). The EMA, however, uses MCIDs to define the clinical relevance of new medicines. The cut-off values found in our study would have consequences for the evaluation of the clinical relevance of new COPD medicines. Multiple new (single-agent) inhalation medicines (aclidinium, glycopyrronium, indacaterol, and umeclidinium) for the treatment of COPD have been approved in Europe in the last decade. According to the EMA assessment of those medicines, roughly 50% of all improvements on FEV_1_, TDI, and SGRQ exceeded the MCID and were thus considered clinically relevant (CHMP, 2009; CHMP, 2012a; CHMP, 2012b; CHMP, 2014). When using the mean cut-off values found in the current study, instead of the MCIDs, none of the improvements on TDI and SGRQ would still have been clinically relevant. A new medicine that is considered “clinically relevant” by registration authorities does, therefore, not necessarily reflect healthcare professionals’ views on clinical relevance. Since healthcare professionals have a stricter view of cut-off values for clinical relevance, defining clinical relevance by use of MCIDs might lead to overestimation of the expected treatment benefit.

Our results indicate that healthcare professionals consider exacerbations as the most important endpoint. Evaluation of the clinical importance of a reduction in exacerbations was not included in this investigation. Although evaluation of exacerbations is also part of the assessment procedure of new COPD medicines, there is no specific MCID used (CHMP, 2009; CHMP, 2012a; CHMP, 2012b; CHMP, 2014). Defining an MCID for COPD exacerbations is problematic, because the impact of exacerbations is influenced dramatically by the used definition of (the severity of) an exacerbation and the influence of baseline status (Chapman et al., 2013). The use of exacerbation-free time instead of frequency (or severity) of exacerbations might better reflect the burden of exacerbations in COPD patients (Boer et al., 2018). Future work should reveal the clinical relevance of a reduction in incidence or severity of exacerbations, or the increase of exacerbation-free time.

This study only included the assessment of the efficacy of new medicines for COPD. This is only a part of the assessment of new medicines, since safety and ease of use are also of clinical importance (CHMP, 2009; CHMP, 2012a; CHMP, 2012b; CHMP, 2014). Our investigation did also not include patient preferences on endpoints and MCIDs. A comprehensive literature review of patient preferences for the management of COPD revealed that the most important issues to patients with severe disease were symptom control, impact of disease on daily life, and positive relationship with the primary caregiver (Bereza et al., 2015). Another study reported the most reported ideal treatment factors based on interviews with 72 patients with asthma or COPD. These patients mentioned improved sleep, speed of action, and length of relief as most important aspects of treatment (Svedsater et al., 2017). Patients perspectives on MCIDs are to our knowledge still unknown.

This investigation was meant as a first study to explore the opinion of different healthcare professionals (physicians, pharmacists, and practice nurses) on clinical relevance of endpoints and cut-off values. Since this study is based on the opinion of healthcare professionals working with COPD patients in daily practice, it provides a clear view of how clinical relevance of new medicines is considered in the daily practice of physicians, pharmacists and practice nurses. The main strength of this investigation is the exploratory and open character which was stimulated by the questionnaire with open-ended answers. There are, nonetheless, some limitations of this study. First, the results cannot be generalized to all Dutch healthcare professionals. Since the IRUM’s CRM was used, only healthcare professionals who were somehow interested in pharmaceutical care were included in this study. Second, approximately half of the responders were pharmacists. In general, they will have less clinical experience than physicians and practice nurses. The overrepresentation of pharmacists could have influenced the mean cut-off values. However, the analysis of the cut-off values per profession showed that the results of the different professions were generally in line with each other. Third, the response rate was poor, with only 6.6 percent response to the general questionnaire and 2.9 percent to the COPD section. The number of responders that completed the questions about cut-off values was even lower. There are some possible explanations for this. Since the healthcare professionals were enrolled in a CRM instead of a research panel, they did not routinely participate in investigations and were not used to be approached for research *via* this panel. Another contributing factor to the low response was the fact that this research was part of a more extensive investigation towards the opinion of healthcare professionals about new medicines. A substantial number of healthcare professionals dropped out before the COPD section. However, unless the poor response rate, there was still a considerable number of healthcare professionals available for analysis. Fourth, the questions about cut-off values for TDI and SGRQ might have been fairly difficult to answer, since these instruments are not routinely used in Dutch clinical practice. This seems to be reflected by the wide range of cut-off values for clinical relevance. To maximize the probability of getting reliable results, the questionnaire referred to the range of scores for TDI and SGRQ. This enabled healthcare professionals unfamiliar with these scales to estimate a clinically relevant difference. However, this does not completely rule out the possibility of inaccuracy in the mentioned cut-off values for TDI and SGRQ.

Despite these limitations, this study suggests that healthcare professionals are more critical than registration authorities in defining the clinical relevance of the efficacy of new medicines for the treatment of COPD. Larger and more representative *ad hoc* trials are needed to focus the topic and confirm these results. In the meantime, the established MCIDs should be used with caution, since new medicines that exceed the MCID do not necessarily meet the expectations of clinical relevance according to healthcare professionals. Defining clinical relevance by using MCIDs might, therefore, lead to overestimation of the expected treatment benefit of new COPD medicines by healthcare professionals.

## Data Availability Statement

The raw data supporting the conclusions of this article will be made available by the authors, without undue reservation, to any qualified researcher.

## Author Contributions

MD and SS designed and conducted the online survey. MD and TS analyzed the data. AL verified the data analysis. MD, MN and LD wrote the first draft. All authors revised the manuscript.

## Conflict of Interest

LD received funding from Astra Zeneca, Abbvie, and Pfizer for studies not related to this study. TS received funding from GlaxoSmithKline, Boehringer Ingelheim, and Novartis Pharma for studies not related to this study.

The remaining authors declare that the research was conducted in the absence of any commercial or financial relationships that could be construed as a potential conflict of interest.
